# New Appliances and Things Medical

**Published:** 1900-05-05

**Authors:** 


### NEW APPLIANCES AND THINGS MEDICAL.

**[We shall be glad to receive, at our Office, 28 & 29, Southampton Street, Strand, London, W.C., from the manufacturers, specimens of all new preparations and appliances which may be brought out from time to time.]**

### IALINE PREPARATIONS.

(Burt, Boulton, and Haywood, Prince Regent's Works, Silvertown, London, E.)

Our representative recently made a tour of inspection through the great works belonging to the above firm, which are situated on the banks of the Thames at Silvertown. Under the able conduct of Mr. E. R. Gabbett, M.I.C.E., the chief engineer; Mr. H. Fergusson, managing chemist; and Mr. R. T. Clark, manager of the antiseptic department, he was enabled to examine in detail the sundry stages of the various processes which are involved in the manufacture of the Ialine antiseptics from the crude coal tar which reaches the works in thousands of tons. From that portion of the distillate of tar which is known as dead oil or creasote, and which, in a more or less crude state, is used in that huge industry, the antiseptic treatment of timber, the refined and purer antiseptics are separated, which are used in surgery and for sanitary purposes.

These consist chiefly of carbolic and cresylic acids together with certain allied chemical bodies. Each of them is independently an antiseptic of recognised value, but a suitable combination of them possesses antiseptic and germicidal properties which are superior to any of them singly. The basis of the Ialine series of preparations is such a combination of antiseptic bodies. It is represented in the solid form by a disinfecting powder containing, among other bodies, 15 per cent. free phenols. This powder is a medium for disinfecting water-closets, drains, traps, and urinals. Of fluids the Ialine series contains several representatives, all of which are miscible with water and contain various percentages of phenols and other antiseptic bodies. That which is known as the Ialine disinfecting fluid can be obtained in three degrees of strength, the "A" fluid, which contains 25 per cent. tar acids; the "C" fluid, 23 per cent.; and No. 2 fluid, 16 per cent. Those containing the higher percentages of acids will stand considerable degrees of dilution, and yet maintain their effectiveness as antiseptics and germicides. They will be found an economical medium for disinfecting extensive areas, such as buildings, trucks, shipholds, &c., which have been exposed to contamination, and other centres of infection. The Ialine sheep dip is a safe, efficient, and rapid medium for the treatment of tick and maggots in these or other animals. The Ialine antiseptic ointment and that which is specially prepared for use in veterinary practice are valuable antiseptic salves for the treatment of wounds, burns, and many varieties of skin disease. Though not strictly belonging to the Ialine series, we may mention certain other antiseptic preparations which are manufactured by the same firm, such as the naptholine and carbolic tablets and soluble and insoluble pine oil blocks, for use in lavatories and in the prevention of insect pests. We have examined samples of these preparations, and have watched the various processes of their manufacture, and can assure readers of The Hospital that they are preparations of the highest class, with the properties which are claimed for them.

### CAMWAL MINERAL WATERS.

(London, Manchester, Birmingham, &c.)

We have examined several samp'es of mineral waters of the above brand. They include syphons of soda water, containing five grains and thirty grains respectively of bicarbonate of soda, potash water with five and fifteen grains bicarbonate of potash, and lithia water manufactured according to B.P. strength. Each variety of water which we have examined was mineralised according to specification, and richly charged with gas. Physicians who wish to be exact in their orders with regard to degree of mineralisation, and the public who care about purity in the manufacture, will do well to make a note of the Camwal brand of mineral waters.

